# Supplementary material for: Offensive language detection in low resource languages: A use case of Persian language
Source: PLoS One. 2024 Jun 21;19(6):e0304166. doi: 10.1371/journal.pone.0304166 (PMC11192314; doi:10.1371/journal.pone.0304166)
Supplement: Attachment — Submitted filename: Response to Reviewers.pdf [file pone.0304166.s002.pdf]

Title: Offensive Language Detection in Low Resource Languages: a use case of Persian language

Authors: Marzieh Mozafari, Khouloud Mnassri, Reza Farahbakhsh, and Noel Crespi

Ref: PONE-D-23-40988

Response to Reviewers' Comments

Dear respected editor and reviewers,

Thank you for the valuable time dedicated to reviewing our manuscript and providing constructive feedback. We sincerely appreciate your insightful comments and suggestions, which have immensely contributed to enhancing the quality of our work. In response to your feedback, we have diligently revised the manuscript. All alterations, including text changes and improvements, have been highlighted in blue within the track changes version for your convenience in identifying the modifications. Please refer to Section 1 for our responses to the minor revisions and Section 2 for those addressing the major revisions.
We trust that these revisions will meet the expectations and significantly enhance the manuscript's quality. Should you require any further clarification or have additional queries, please do not hesitate to contact us.
Additionally, we thoroughly reviewed the points outlined in the journal requirements, which are detailed in Section 3 of this rebuttal. In our revised version, we ensured to address these points comprehensively.
Thank you once again for your time and valuable input. We look forward to your feedback on the updated version of our paper.

Best Regards,
Khouloud Mnassri

Section 1: Minor revisions

Contribution: compare many models, present new architecture in hope of solving the problem with improved performance and an experiment. Persian Dataset annotation

Relevant literature: adequately cited and discussed w.r.t different languages and different models for the chosen task. However choices are only cursorily supported by literature

Presentation: good

Clarity: fair enough

Organization: fair enough(most revisions relate to this)

Reproducible results: yes, model seem adaptable

Purpose of model: well described/ supported by literature

Input/output and internal processing: fairly described

Illustrations: well made

Answer:
We are grateful for your careful reading of our work and insightful feedback. We value your compliments regarding our work, especially the way we introduced a new architecture and compared several models in the field. We are glad to learn that our manuscript's clarity and presentation meet your expectations and that our models appear adaptable. We also appreciate your acknowledgment of the goodness of our illustrations.

Minor Revisions comments:

1. References are not an alphabetically ordered list

Answer:
We followed the Plos One template for the text and references.

2. The English of write-up can be improved overall, headings should be in sentence case and punctuation marks at correct places.

Answer:
Thank you for your valuable feedback. We have thoroughly reviewed the manuscript, and we have made all the required corrections to enhance the English language writing, including handling spelling mistakes and assuring proper punctuation. Additionally, we have revised the headings to conform to the sentence case.

Action:
In the revised manuscript, corrected letters, words and expressions, and punctuation marks are underlined in blue.

3. The inclusion of web links in text is not a good idea, add in footnotes or references

Answer:

Thanks for this remake. We found out in the Plos One template it is not allowed to add footnotes. However, we fixed this point in the revised version based on your comment.

Action:

We've revised the manuscript accordingly by relocating all web links to footnotes.

4. All the sections and sub sections have to be numbered, right from beginning with introduction to conclusion

Answer:

We followed the Plos One template for building the sections and subsections of our manuscript.

5. There is no discussion about future recommendations , not even a single line in the last section

Answer:

Thank you for pointing this out. Within the 'Conclusion' section, we have provided a paragraph outlining future recommendations that we intend to pursue. We have augmented this content with additional insights to further refine our future goals.

Action:

We have highlighted the "Future direction" part at the end of the "Conclusion" section of our manuscript adding a title ("Future directions:") in bold. We added more content to this part as follows:

"In future work, we aim to deal with our imbalanced dataset by leveraging textual data augmentation techniques for Persian language, using various techniques like generative GPT3 PLM-based models ([75]) which were used on English data, but we believe we can employ it for low-resource languages like Persian. We aim to train more complex DL and Large Language models even in a multilingual configuration to deal with English-Persian code-mixing offensive language content on online social media. We are also eager to use our dataset to train on the recently released Large Language Models in order to learn more about their efficacy and generalizability, which will improve our comprehension and application of these innovative technologies.

Furthermore, we believe that considering linguistic-based characteristics of offensive language in Persian as a low-resource language would give more precise results in detecting such content on social media."

6. Add percentage of achieved improvement in conclusion section

Action:

Thank you for your valuable feedback. We consider your comment and add content in the "Conclusion" section based on it. The new content is as follows, where we underlined the added text:

"We got outstanding performance results on each of the three levels of annotation, getting macro F1 score of about: 90% using SVM Word n-grams model, 89.6%, and 82% using SVM Character n-grams model, on each of the first, second and third model respectively.

Furthermore, we built an ensemble stacking model to increase the performance of the classification task by selecting the least correlated single classifiers with different skills on the problem of offensive language detection. The results signify that among single models, the SVM model trained on character or word n-grams followed by pre-trained monolingual model ParsBERT performs the best in the identification of offensive vs non-offensive content, targeted vs untargeted offensive content, and targeted offensive content towards individual or groups in almost all cases. Furthermore, using an ensemble stacking model results in increasing the F1-score of the classification task over single classifiers with a performance improvement of about 5%."

7. Wherever mention number of instances of dataset, use complete number: 520,000 instead of 520k

Action:

Thank you for pointing this out. We fix the number of instances as per your remark in our manuscript.

8. At the end of introduction section , a paragraph that describes the organization of the rest of the paper is required (outline of the study)

Action:

We appreciate your valuable feedback. We have added this paragraph at the end of the 'introduction' section in order to outline clearly the structure of our study. The text is as follows:

"In the next section titled 'Related Work,' we will go through existing studies on offensive language detection techniques, examining both English and other languages, specifically low-resource ones and the Persian language. Following this, in the 'Dataset' section, we will present a comprehensive overview of the creation of our dataset, beginning with the measures taken for data collection, and then, describing the data annotation procedure. Subsequently, in the 'Methodology' section, we will present the methodology of our extensive experiments. This will involve describing the various models we plan to employ, clarifying the experiments executed, and concluding with the presentation of results and our analysis. Finally, we will present our conclusions and summarize future recommendations for further investigation in the 'Conclusion' section."

9. Main contributions in the introduction section has to be numbered list rather than bullet points

Action:

Thank you for your comment. We changed the contributions in the 'Introduction' section into numbered list as requested.

The added text is as follows:

"1. Building and sharing Persian offensive language corpus along with describing the methodology for collecting tweet data from X and annotation guidelines.

2. Performing comprehensive experiments on annotated Persian corpus to investigate the ability of classical ML, DL, and transformer-based neural network models in addressing Persian offensive language identification task in social media.

Furthermore, we focus on transfer learning approach using advanced monolingual

and multilingual pre-trained language models such as ParsBERT, ALBERT-Persian, mBERT, and XLM-RoBERTa for Persian offensive language detection task.
3. Introducing a stacked ensemble methodology to improve the performance of the proposed offensive language detection models.
In the next section titled ‘Related Work,’ we will go through existing studies on offensive language detection techniques, examining both English and other languages, specifically low-resource ones and the Persian language. Following this, in the ‘Dataset’ section, we will present a comprehensive overview of the creation of our dataset, beginning with the measures taken for data collection, and then, describing the data annotation procedure. Subsequently, in the ‘Methodology’ section, we will present the methodology of our extensive experiments. This will involve describing the various models we plan to employ, clarifying the experiments executed, and concluding with the presentation of results and our analysis. Finally, we will present our conclusions and summarize future recommendations for further investigation in ‘Conclusion’ section.”

10. The figure elaborating the research methodology needs revision,

- like in preprocessing module all steps need to be connected (right now they looked stacked),
- also inclusion of input and output labels/evaluation methods is nowhere except mentioning that tweets are the input,
- also there is no mention of datasets in the figure

Answer:
Thank you for your insightful comment. We acknowledge the importance of having a clear and comprehensive figure to display the workflow of our methodology in detecting offensive language and hate speech in Persian. We have carefully considered your statements and have made the necessary adjustments to the related figure (Figure 1) as per your request.

Action:
We have included several modifications to the corresponding figure to enhance clarity and comprehension. In the preprocessing part, we introduced arrows to indicate the sequential nature of the steps and to order them as we mentioned in the next sections. Additionally, we have also enclosed a separate figure (Figure 5) particularly dedicated to visualizing our preprocessing step in their sequential order, providing further clarity.

To enhance understanding, we have labeled the dataset as **"Dataset"** within the figure, emphasizing that it’s created from tweet samples collected from "X" (or Twitter platform), this is the input of our models, which seems obvious, so we didn’t highlight it as input. As for the output, it is the classification results of the dataset classes (annotations), which are elaborated upon in the "annotation schema" section of the figure.

We aimed to keep simplicity in the figure, as it serves as a general overview of our work at the beginning of the paper. Detailed explanations of each element of the figure will be provided in subsequent sections, ensuring comprehensive understanding for the reader.

Please find the updated version of the figure below:

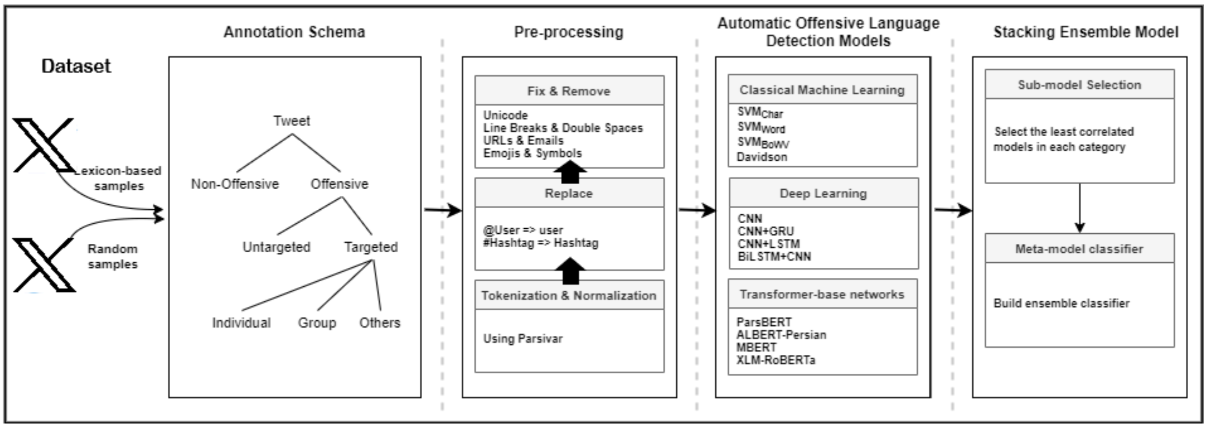

11. add separate tables for **names of models** involved in various sub sections of methodology section, along with their **parameters** and **specs**

Answer:

Thank you for your feedback. We anticipate that these modifications will enhance the clarity of our paper, making it more readable and comprehensible.

Table 5 in our manuscript, already gives details about the “Monolingual and multilingual transformer-based networks” we used in our experiments, so we will present the tables of the other models, namely the baselines (ML and DL models). As for the stacking ensemble, we already explain the model algorithm in which we consider using SVM as a meta-level classifier we trained to get the output final results.

Action:

We have included additional tables following each category of models: baseline, Monolingual and multilingual transformer-based models, and stacking ensemble models. In Tables 4, 5we provide detailed information on the parameters and characteristics of each model, as indicated in your valuable comment.

ps: we cannot highlight the tables content all in blue, therefore we color the tables titles to highlight the Tables we add upon your comments.

- For Table 3, we added this text content to introduce the table, “To summarize the baseline classical machine learning models, we added Table 3. It includes parameters, feature extraction techniques, and the specifications of each model.”

Table 3. Baselines ML models.

Model name	Feature extraction method	Parameters	Characteristics
$SVM_{Character\ n\text{-}grams}$	TF-IDF on character n-grams	logistic regression with L1 regularization	n = 2<br>n = 5
$SVM_{Word\ n\text{-}grams}$	TF-IDF on word n-grams		n = 1<br>n = 2
SVM_{BoWV}	BoWV over fastText - accompanying Davidson algorithm		fastText with an embedding dimension of 300<br>Logistic regression with L2 regularization

- For Table 4, we added this content: “We have added all the deep learning models we used in Table 4, including feature extraction method, parameters and specifications of each model.”

Table 4. **Baselines DL models.**

Model name	Feature extraction method	Parameters	Characteristics
CNN	fastText representation	Softmax activation function<br>binary-crossentropy loss function<br>Adam optimizer.	1D convolutional layer<br>with 64 filters and a window size of 4<br>pooling layer<br>max pooling layer with a pool size of 2<br>ReLU activation function
CNN+BiLSTM	fastText representation	Softmax activation function<br>binary-crossentropy loss function<br>Adam optimizer.	drop-out layer with a rate of 0.2<br>bi-LSTM layer with 128 units<br>1D convolutional layer with 100<br>filters with a window size of 2<br>Average pooling<br>Max pooling<br>dense layer with 64 units<br>ReLU activation function
CNN+GRU<br>and<br>CNN+LSTM	fastText representation	Softmax activation function<br>binary-crossentropy loss function<br>Adam optimizer.	drop-out layer with a rate of 0.2<br>1D convolutional layer with 100<br>filters with a window size of 4<br>ReLU activation function<br>1D max pooling layer with a pool size of 4<br>global max pooling layer

12. Can I ask why this split of 90-10? I mean with such copious amount of data, is it needed? Is it not too biased split?

Answer:

Thank you for the inquiry. The decision to adopt a 90-10 split for training and validation was carefully considered to strike a balance between leveraging a substantial dataset for model training while ensuring robust validation. We already split the dataset, which is of 6000 samples size into 80-20 split for training - testing subsets. This is very common in fine tuning ML models. Then, we took the training set (which is the 80% of the whole 6000 samples data) and allocate 10% of the training set as a validation set for hyper-parameter tuning (thus we got this 90-10 split: 4320 samples for training and 864 for validation). We explained this in this text part: “For classical ML models, we split data to train and test sets by 0.8 and 0.2. To train DL models and fine-tune other models based on monolingual or multilingual pre-trained language models, we consider 0.1 of the train set as a dev set for hyper-parameter tuning.”

Given the relatively modest size of our dataset, comprising 6000 samples, reserving 10% for validation (out of 80% of the whole training data) enables a thorough examination of the model's generalization performance and helps determine potential overfitting. Additionally, we utilized k-fold cross-validation (k = 5), which necessitates plenty data samples to ensure each fold has sufficient data for robust evaluation. Specifically, each fold in the k-fold cross-validation consisted of a train set with 3456 samples, a validation set with 864 samples, and an unchanged test set with 1200 samples. We've also taken steps to mitigate bias concerns by ensuring random data splitting.

13. Preprocessing section contains some vague text in language other than English

Answer:

Thank you for pointing out this matter. We have removed this text from the preprocessing section. It appears that this paragraph was part of the PLOS ONE template and was

inadvertently included. We apologize for any confusion this may have caused and thank you for bringing it to our attention.

14. A diagram of data annotation process can be added to illustrate it in dataset section
Answer:
Thank you for your suggestion. We believe that adding a diagram of preprocessing steps would indeed enhance the clarity in our manuscript. As a result, we have included this diagram below (Figure 5), accompanied by the text:
"Overall, Figure 5 provides a clearer visualization of these preprocessing steps."
This is the Figure we added.

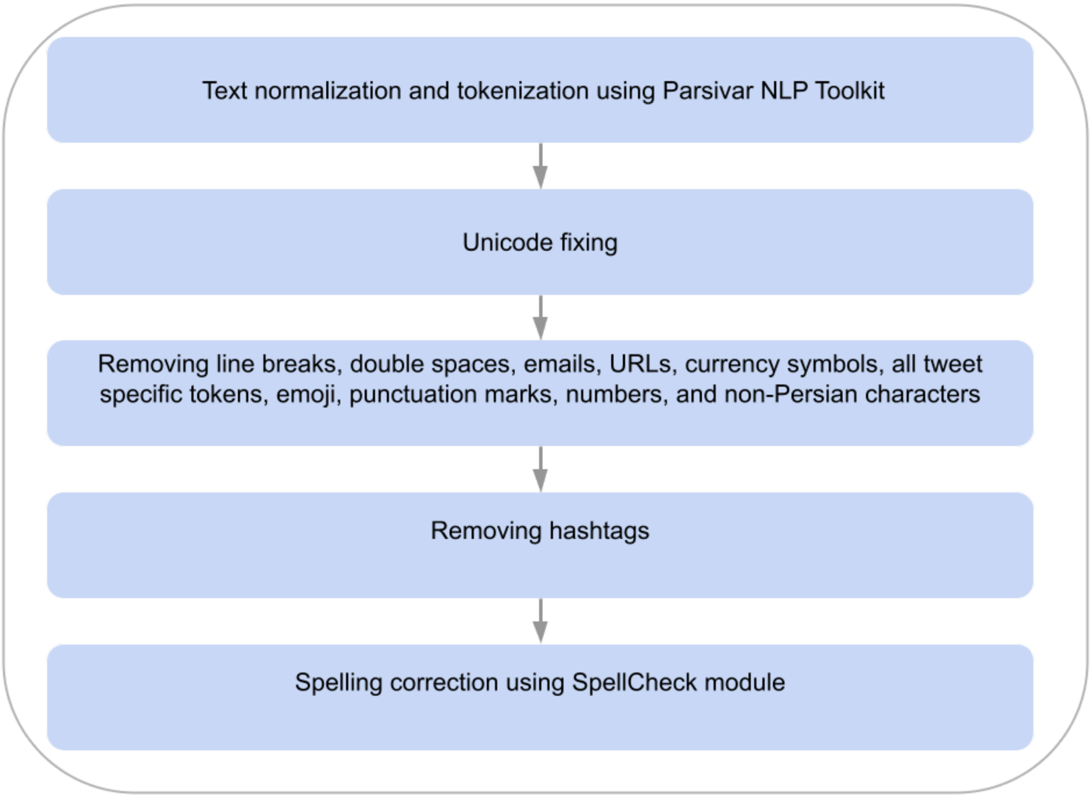

15. A diagram of stacking ensemble's working, as given by the algorithm, is needed
Answer:
Thank you for your feedback. We acknowledge the importance of providing a visual representation to enhance the comprehensibility of the stacking ensemble algorithm. Consequently, we have included a diagram illustrating the workings of the stacking ensemble learning algorithm in our manuscript. This addition aims to facilitate a clearer understanding of the method for our readers.

Action:
We added the diagram of the stacking k-fold cross validation algorithm in Figure 4, along with the following text: "To give a more visual presentation of the stacking ensemble learning algorithm we used, we added the diagram in Figure 4."

This is the figure we added:

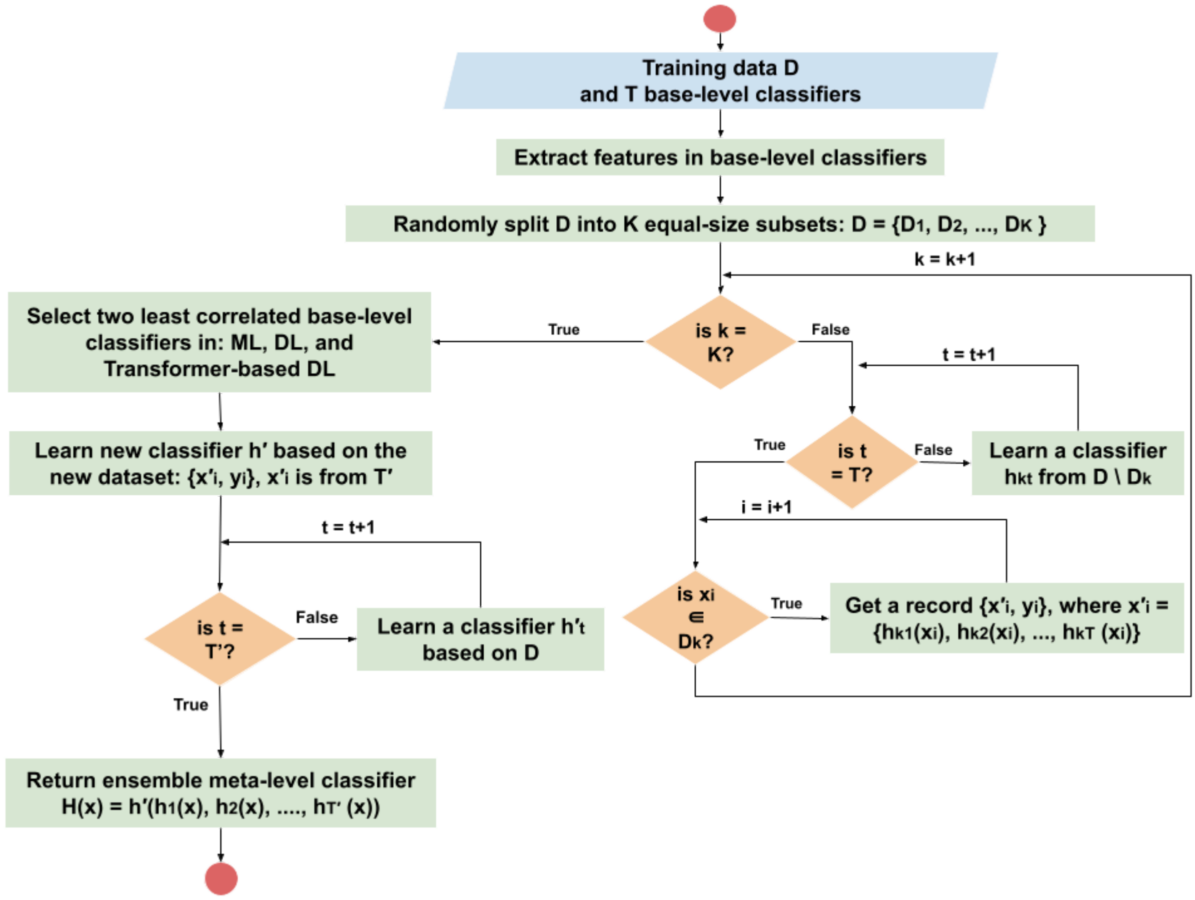

16. the algorithm need line numbers in it and should be explained with reference of line numbers in the paragraph above it.

Answer:

Thank you for your insightful feedback. We greatly appreciate your suggestion regarding the addition of line numbers to the algorithm, as it facilitates easier referencing of specific sections. Accordingly, we have implemented line numbering in the algorithm and referenced them in the text above it.

Action:

Incorporating the line numbers into the algorithm enables us to easily reference each part of the code in alignment with our preceding description of the algorithm. The updated content is as follows:

"The stacking ensemble algorithm is summarized in Algorithm 1. First, we extract all features for different classical ML models (line 1) and prepare the input of DL and transformer-based neural networks according to previous subsections (line 2 - line 13). Given the labeled dataset, we use a k-fold cross-validation approach on the entire data to learn base-level classifiers separately (line 5 - line - line 8) and use their out-of-fold predictions as training features for meta-level classifiers (line 9 - line 12)."

17. There has to be a clear mention of the evaluation metrics to be used in the methodology or experiment section (right now its slightly mentioned in the start of results section)

Answer:

Thank you for your valuable feedback. We are aware of the importance of clearly defining the evaluation metrics in the methodology or experiment section. We understand that the current placement of this information at the beginning of the results section may not provide the necessary clarity regarding our performance evaluation.

To address this concern, we added content in the experiment section by incorporating a detailed explanation of the evaluation metrics to be used. Specifically, we will emphasize the significance of Precision (P), Recall (R), and F1-score (F1) per class, along with the macro-averaged F1 score, in assessing the performance of our models.

Action:

In the experiment section, we provided a comprehensive overview of the metrics used at the end of the section in “Evaluation metrics” part.

Section 2. Major revisions Reviewers' comments:

1. Is the manuscript technically sound, and do the data support the conclusions?

The manuscript must describe a technically sound piece of scientific research with data that supports the conclusions. Experiments must have been conducted rigorously, with appropriate controls, replication, and sample sizes. The conclusions must be drawn appropriately based on the data presented.

Reviewer #1: Yes
Reviewer #2: Yes
Reviewer #3: Partly

---

2. Has the statistical analysis been performed appropriately and rigorously?

Reviewer #1: Yes
Reviewer #2: Yes

Reviewer #3: N/A

---

3. Have the authors made all data underlying the findings in their manuscript fully available?

The PLOS Data policy requires authors to make all data underlying the findings described in their manuscript fully available without restriction, with rare exception (please refer to the Data Availability Statement in the manuscript PDF file). The data should be provided as part of the manuscript or its supporting information, or deposited to a public repository. For example, in addition to summary statistics, the data points behind means, medians and variance measures should be available. If there are restrictions on publicly sharing data—e.g. participant privacy or use of data from a third party—those must be specified.
Reviewer #1: Yes
Reviewer #2: No
Reviewer #3: Yes

---

4. Is the manuscript presented in an intelligible fashion and written in standard English?

PLOS ONE does not copyedit accepted manuscripts, so the language in submitted articles must be clear, correct, and unambiguous. Any typographical or grammatical errors should be corrected at revision, so please note any specific errors here.

Reviewer #1: Yes
Reviewer #2: Yes
Reviewer #3: Yes

---

5. Review Comments to the Author

Please use the space provided to explain your answers to the questions above. You may also include additional comments for the author, including concerns about dual publication, research ethics, or publication ethics. (Please upload your review as an attachment if it exceeds 20,000 characters)

Reviewer #1: Contribution: compare many models, present new architecture in hope of solving the problem with improved performance and an experiment. Persian Dataset annotation
Relevant literature: adequately cited and discussed w.r.t different languages and different models for the chosen task. However choices are only cursorily supported by literature
Presentation: good
Clarity: fair enough
Organization: fair enough(most revisions relate to organization of paper)
Reproducible results: yes, model seem adaptable
Purpose of model has been well described/ supported by literature
Input/output and internal processing have been fairly described
Illustrations are well made

Answer:
Thank you for your detailed review of our work. We appreciate your positive feedback concerning the presentation, clarity, and organization of the paper. Your recognition of the reproducibility of our results and the thorough description of our model's purpose and procedures are particularly encouraging.
We also extend our appreciation for your favorable comments on the illustrations included in the paper. We believe that clear visual illustrations have a vital role in enhancing understanding.

We also value your feedback on our literature review section. We want to confirm that we have provided a comprehensive review of the relevant literature.

1. Our focus has been on offensive language and hate speech detection techniques, particularly highlighting and presenting existing approaches that use machine learning (ML), deep learning (DL), and transfer learning (TL).
2. Furthermore, we have paid attention to multilingual approaches in this domain, highlighting the languages used and the methodologies employed for each one.
3. In addition, we have dedicated a subsection to “language-specific abusive language detection”, where we have summarized (in Table 1) recently shared tasks and academic events addressing various languages in the field and providing several corpora in different languages.
4. We have also provided detailed insights into approaches focusing on low-resource languages, explaining the methodologies used and the creation process of datasets, when they exist.
5. Moreover, we have specifically delved into the domain of hate speech and offensive language detection in the Persian language, meticulously presenting existing studies along with details on dataset creation, including size and source, as well as the machine learning models and feature extraction techniques utilized.

Overall, we believe our literature review effectively covers the existing studies on hate speech and offensive language detection. We've taken a thorough strategy,

examining these studies from numerous angles, including sequentially their **general** scope, **multilingual** concerns**,** attention to **low-resource languages**, and then an observant investigation of **Persian language** studies. Moreover, we've provided an extensive overview of shared tasks and events, as they also present significant sources of datasets in the field. Our review proposes detailed insights into the datasets created through recent shared tasks, including the topic addressed (toxicity, misogyny…), languages illustrated, sample sizes of corpora for each language, and the platforms from which data was sourced (Twitter, Facebook…). Through this detailed literature review, we aim to provide readers with a good insight of hate speech and offensive language detection research, while always highlighting the various range of datasets available and created. Overall, we aimed not to delve deeply into each approach in our 'Related work' section. We believe that this would especially expand the content and risk overwhelming the reader. Instead, our goal is to offer the literature from a simplified perspective, highlighting inspiration and the gaps we plan to address within our paper.

Reviewer #2: In Iran do not could access Twitter without restrictions and can access Twitter with VPN.
Persian-speaking Twitter users are mostly outside Iran who have migrated for political reasons, and considering that the vast majority of offensive examples are related to political parties and government issues of Iran, offensive words have been used offensive words by these users.
The second group of Twitter users are Iranian authorities who cannot use offensive words in their tweets.
Other people don't have access to Twitter without VPN and it is not a popular network like Instagram in Iran.
Therefore, the sampling used should be Cluster sampling.

Answer:
Thank you for your insightful remark regarding the sampling procedure used in our study. We are aware that Twitter access in Iran is restricted, and we acknowledge that Persian-speaking Twitter users, are mostly outside Iran.
In our study, we used **random sampling** and **lexicon-based sampling** methods for data collection. However, we realize that these methods may not fully catch the perspectives of all Twitter users in Iran, particularly considering the limitations you've underlined regarding access and user demographics.

Therefore, we have addressed this as a limitation of our data collection part in our study in the 'Dataset' section, emphasizing the likely bias presented by the sampling methods employed. Additionally, we introduce cluster sampling as an alternative strategy that may offer enhanced representativeness and mitigate biases during data sampling.

Action:

We have added the following text in the ‘Dataset’ section at the end of the ‘Data collection’ subsection:

*“**Limitations of data collection - data sampling:***
It is important to acknowledge the restrictions associated with the sampling techniques used for data collection. We employed random sampling and lexicon-based sampling techniques to collect tweets related to Persian hate speech and offensive language on Twitter. However, we acknowledge that these procedures may present bias and may not fully capture the perspectives of all our Twitter users. As a result, the nature of our sampling approach may not give the best representation of certain user groups. This limitation could eventually affect the generalizability of our findings. We aim in the future to address these limitations by analyzing alternative sampling methods, such as cluster sampling, to enhance the representativeness of our data and mitigate biases associated with user demographics and access constraints.”

What is the reason for not using other machine learning methods in this manuscript?
Answer:
Thank you for your inquiry about the choice of machine learning techniques in our manuscript. We have focused on a specific set of well-established techniques that have been extensively employed and validated within the research studies for hate speech and offensive language detection tasks. While we recognize the existence of alternative machine learning methods, we have previously examined some of these techniques in Persian language in a separate study, particularly concentrating on meta-learning models such as MAML and Proto-MAML for cross-lingual few-shot hate speech and offensive language detection (we mentioned this work in our manuscript). Our decision to work on the chosen methods in this manuscript was inspired by a comprehensive literature review of previous studies on hate speech and offensive language detection (in the section “Related work”): in general, multilingual, low-resource languages, and in the Persian language. Inspired by the methods outlined in these studies, we chose the most commonly used ones, considering their demonstrated effectiveness in the field.

Moreover, within the “Conclusion” section of our paper, we have highlighted our aim to explore more complex deep learning models and Large Language Models (LLMs) in our future directions part. We believe that these advanced models would further help enhance the performance of our detection tasks in the Persian language.

Reviewer #3: The manuscript's structure is confusing, making it challenging to comprehend the improvements made in comparison to previous studies. The authors should provide a clear description of their enhancements for better understanding.
Answer:
Thank you for your valuable feedback. In reply to your comment regarding the manuscript's structure, we have made the inclusion of a diagram presenting the paper's structure, which is very much classic and similar to the standard papers structure in the NLP machine learning field. This diagram delivers a visual overview of the different sections in our study, from examining related work and presenting our dataset creation to detailing our models building, as well as the training process, and we ended up illustrating results and analysis. We believe

that this visual diagram will greatly assist readers in comprehending the flow and organization of our study.

Regarding the enhancements of our work, in our ‘Related work’ section, we determined the gaps in the academic research concerning offensive language detection in Persian due to the lack of annotated datasets in the field. Our work addresses this gap by collecting tweet data from Twitter (now known as the ‘X’ platform) and executing annotation practices to construct a comprehensive 3-annotation level hateful and offensive language corpus in Persian. Then, we used our datasets to operate extensive experiments comparing classical ML, DL, and transformer-based models, including ParsBERT, ALBERT-Persian, mBERT, and XLM-RoBERTa, to assess their performance and the improvements they add in the fine-tuning results (as we described in ‘Results and Analysis’ subsection). Additionally, we introduce a stacked ensemble methodology to enhance further the overall effectiveness of these models which yielded better results as we described in our manuscript.

We believe that our publicly available dataset will serve as a useful resource for researchers interested in hate speech and offensive language detection in Persian language. Moreover, by delivering performance results of various supervised learning techniques, most commonly used in hate speech and offensive language detection, including ML, DL, and Transfer Learning, our study provides readers with a comprehensive overview of the methodologies in the Persian language in this field. Our work not only enables access to essential data but is also considered a guideline for anyone seeking detailed knowledge of different supervised learning approaches within this domain.

Action:
To make it clear, we added this figure at the end of the ‘Related work’ section, presenting our paper structure, the text is as follows:
“For a better understanding of the structure of our study, we present a diagram
(Figure 2) proposing the flow of our paper. This structure shows the main steps we took
during our study from literature review to data creation, models development and
training, finishing with results and analysis. “

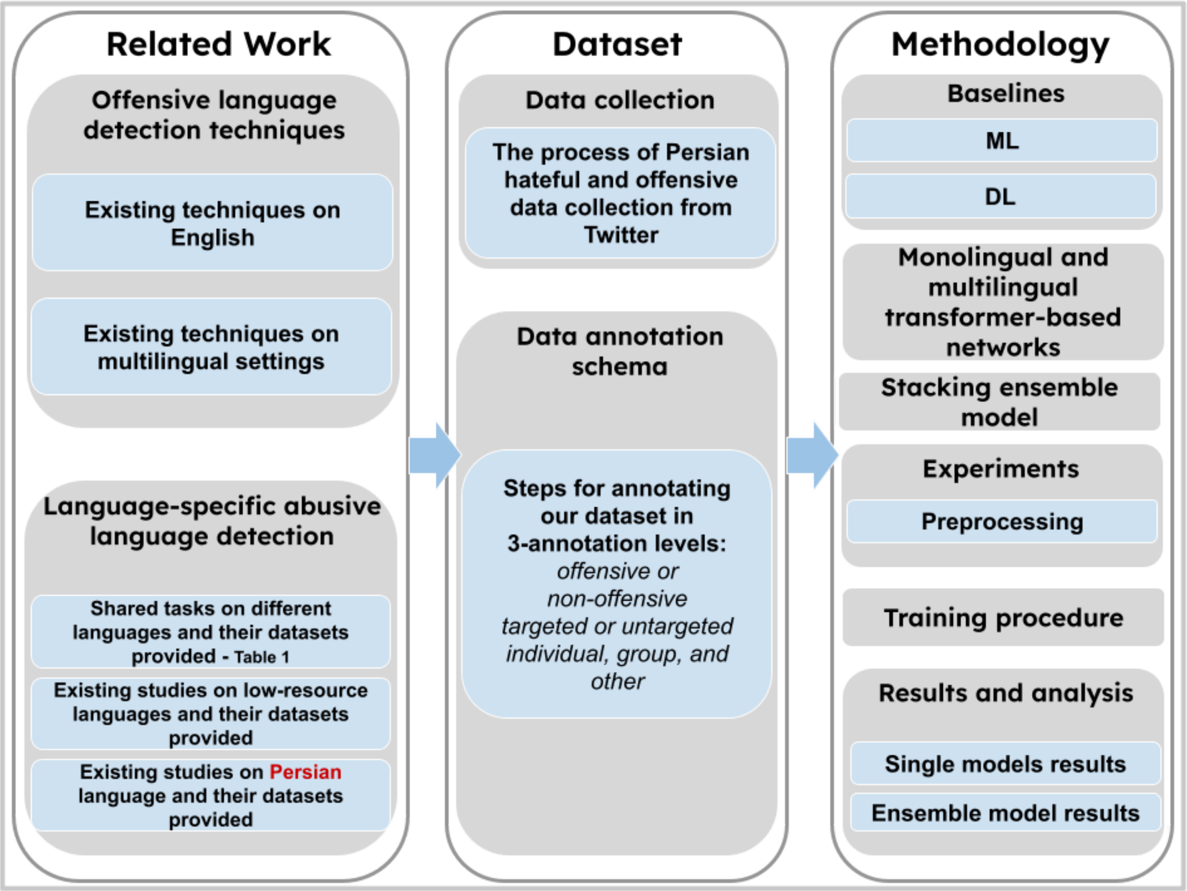

The manuscript would benefit from the inclusion of a figure to elucidate the proposed method, enhancing the clarity of the methodology.

Answer:

Thank you for your comment. We have already incorporated in our manuscript: "Fig 1. Workflow of the offensive language detection methodology in Persian language" to visually introduce our proposed approach. This figure includes every step we took in our study, from dataset collection and annotation to data preprocessing, followed by illustartion of our detection models and the stacking detection model. We believe this figure offers a clear and comprehensive presentation of our methodology, providing readers with a simplified yet detailed overview of our work.

The structure of the manuscript appears monotonous. Introducing figures and charts would alleviate this issue, making the content more engaging and accessible to readers.

Answer:

Thank you for your insightful remark. We've made enhancements to our manuscript's structure by including various figures to enhance its visibility and comprehension.

In addition to the existent Figures:

- Figures 1 (giving the workflow of our methodology, the annotation schema of our dataset, as well as all the models' names we used in our experiments),
- Figure 3 (proposing some of our dataset samples in Persian and their translation into English),

- Figure 6 (presenting Correlations between the predicted probabilities of different classifiers on an out-of-fold test set data),
- and Figure 7 (providing the models performance in the three annotation levels of our dataset),

we've added several other Figures to enhance the presentation of our paper.

Our updated version now contains Figure 2, presenting to the readers a clear detailed overview of the paper's structure. This diagram helps to get a better understanding of each section and subsection of our manuscript, giving an overall general comprehension. Furthermore, we've enclosed Figure 4, offering a simple presentation of our stacking k-fold cross-validation algorithm for easier understanding and interpretation. Also, we added Figure 5, which clarifies the preprocessing steps employed, yielding in better insight into our dataset preparation process.

The author references Ensemble Model Results, but there is a lack of explanation. It is crucial to describe what the Ensemble Model is, providing a clear understanding. Additionally, the inclusion of mathematical equations would be beneficial in elucidating the model's workings.

Answer:

Thank you for your feedback. We have implemented only one ensemble model, which is the stacking ensemble learning model. This model is considerably defined and described in the 'Stacking ensemble model' subsection of our manuscript. Within this subsection, we have offered a detailed illustration and explanation of the ensemble model's workings, including its algorithm and how it's executed in our methodology to improve the performance of our models. Additionally, we have incorporated a diagram to further facilitate the understanding of the algorithm in a visual presentative way.

In response to the suggestion for including mathematical equations, we have taken your feedback into consideration. While our provided algorithm thoroughly describes the k-fold stacking ensemble learning technique we used in our work, we believe that also incorporating mathematical equations would further elucidate the workings of our models. Consequently, we have included the mathematical equations at the end of the 'Stacking ensemble model' subsection. These equations closely reflect the different steps outlined in the algorithm, providing readers with a deeper understanding of the model's operation.

Action:

We have addressed your feedback by explicitly referencing the 'Stacking ensemble model' subsection in the 'Ensemble model results' subsection, where the ensemble model is thoroughly clarified. The text added is as follows:

"The ensemble learning model utilized in this context is outlined in the 'Stacking ensemble model' subsection, where we offer a thorough explanation of our implementation approach for this ensemble algorithm."

We have also added at the end of 'Stacking ensemble model' subsection mathematical equations. Each one is explicitly related to the corresponding steps in our algorithm.

The added text is as follows:

“In order to facilitate further the comprehensibility of the k-fold stacked ensemble learning algorithm we used in our methodology, we provide a general overview of the mathematical presentation of this ensemble learning technique. In fact, as indicated in its algorithm, in stacking with k-fold cross-validation, the procedure involves numerous steps, including training the base-level models on the folds of the data, producing predictions for the validation fold, and then using these predictions as inputs to train the meta-level classifier. The mathematical presentation of this process is as follows, in which we highlighted the corresponding steps of the algorithm:
1) Base-level models training: (steps 1 - 2) [...]
2) Generate Predictions - Construct training set for the meta-level classifier: (step 2.2) [...]
3) After selection of the least-correlated base-level classifiers, and getting T’ base-level classifiers (step 3), we go to:
4) Stacking: (Steps 4 - 5) [...]
We finally get H as trained meta-classifier.”

---

6. PLOS authors have the option to publish the peer review history of their article (what does this mean?). If published, this will include your full peer review and any attached files.

If you choose “no”, your identity will remain anonymous but your review may still be made public.

Do you want your identity to be public for this peer review? For information about this choice, including consent withdrawal, please see our Privacy Policy.
Reviewer #1: Yes: Sadia Tariq
Reviewer #2: No
Reviewer #3: Yes: Saman Forouzandeh

Section 3. Journal Requirements

Journal Requirements:

When submitting your revision, we need you to address these additional requirements.

1. Please ensure that your manuscript meets PLOS ONE's style requirements, including those for file naming. The PLOS ONE style templates can be found at https://journals.plos.org/plosone/s/file?id=wjVg/PLOSOne_formatting_sample_main_body.pdf and https://journals.plos.org/plosone/s/file?id=ba62/PLOSOne_formatting_sample_title_authors_affiliations.pdf

2. We suggest you thoroughly copyedit your manuscript for language usage, spelling, and grammar. If you do not know anyone who can help you do this, you may wish to consider employing a professional scientific editing service.

Upon resubmission, please provide the following:
● The name of the colleague or the details of the professional service that edited your manuscript
● A copy of your manuscript showing your changes by either highlighting them or using track changes (uploaded as a *supporting information* file)
● A clean copy of the edited manuscript (uploaded as the new *manuscript* file).

3. Please note that PLOS ONE has specific guidelines on code sharing for submissions in which author-generated code underpins the findings in the manuscript. In these cases, all author-generated code must be made available without restrictions upon publication of the work. Please review our guidelines at https://journals.plos.org/plosone/s/materials-and-software-sharing#loc-sharing-code and ensure that your code is shared in a way that follows best practice and facilitates reproducibility and reuse.

4. In your Methods section, please include additional information about your dataset and ensure that you have included a statement specifying whether the collection and analysis method complied with the terms and conditions for the source of the data.

Answer: We added ethical consideration part dedicated for this in the paper.

5. You indicated that ethical approval was not necessary for your study. We understand that the framework for ethical oversight requirements for studies of this type may differ depending on the setting and we would appreciate some further clarification regarding your research. Could you please provide further details on why your study is exempt from the need for approval and confirmation from your institutional review board or research ethics committee (e.g., in the form of a letter or email correspondence) that ethics review was not necessary for this study? Please include a copy of the correspondence as an "Other" file.

Answer: We have already addressed the ethical considerations in the "Ethical Considerations" part of our paper. We assure you that we have taken appropriate measures to maintain privacy and ethical standards. Specifically, we collected only publicly available tweets from Twitter (X platform) accounts and anonymized user information to ensure tweeter privacy and GDPR requirements. Additionally, we mentioned that will publish an open dataset without disclosing tweet text or user information (only Tweet ID nad its label). We hope this clarification is clear. Please let us know if you require any further information.

7. PLOS ONE does not copy edit accepted manuscripts. Therefore, the language in submitted articles must be clear, correct, and unambiguous (http://journals.plos.org/plosone/s/criteria-for-publication#loc-5). We notice that your manuscript contains offensive terms in Fig. 2. We do not think that this text is essential to the main manuscript of your study, and we ask you to move Fig. 2 to a Supporting Information file referenced in the main text.

Answer: We placed this figure (which is now Figure 3) in the 'Supporting information' section after the 'Conclusion' section in the manuscript. Its text reference is still in its original place in the manuscript, we only changed the Figure placement.

Please add the following text to the beginning of your Abstract section: "THIS ARTICLE USES WORDS OR LANGUAGE THAT IS CONSIDERED PROFANE, VULGAR, OR OFFENSIVE BY SOME READERS."

Answer: We added this text.

Please also add the following text to the beginning of your Introduction section: "This article uses words or language that is considered profane, vulgar or offensive by some readers. Due to the topic studied in this article, quoting offensive language is academically justified but we nor PLOS in no way endorse the use of these words or the content of the quotes. Likewise, the quotes do not represent the opinions of us or that of PLOS, and we condemn online harassment and offensive language."

Answer: We added this text, replacing the disclaimer we have in the paper.

We appreciate your attention to these requests.

8. We note that your Data Availability Statement is currently as follows: "All relevant data are within the manuscript and its Supporting Information files."

Please confirm at this time whether or not your submission contains all raw data required to replicate the results of your study. Authors must share the “minimal data set” for their submission. PLOS defines the minimal data set to consist of the data required to replicate all study findings reported in the article, as well as related metadata and methods (https://journals.plos.org/plosone/s/data-availability#loc-minimal-data-set-definition).

For example, authors should submit the following data:

- The values behind the means, standard deviations and other measures reported;

- The values used to build graphs;

- The points extracted from images for analysis.

Authors do not need to submit their entire data set if only a portion of the data was used in the reported study.

If your submission does not contain these data, please either upload them as Supporting Information files or deposit them to a stable, public repository and provide us with the relevant URLs, DOIs, or accession numbers. For a list of recommended repositories, please see https://journals.plos.org/plosone/s/recommended-repositories.

If there are ethical or legal restrictions on sharing a de-identified data set, please explain them in detail (e.g., data contain potentially sensitive information, data are owned by a third-party organization, etc.) and who has imposed them (e.g., an ethics committee). Please also provide contact information for a data access committee, ethics committee, or other institutional body to which data requests may be sent. If data are owned by a third party, please indicate how others may request data access

Answer: We confirm that our submission contains all raw data required to replicate the results of our study. We used public dataset, and we reffer it as available data.

9. Please provide additional details regarding participant consent. In the ethics statement in the Methods and online submission information, please ensure that you have specified (1) whether consent was informed and (2) what type you obtained (for instance, written or verbal, and if verbal, how it was documented and witnessed). If your study included minors, state whether you obtained consent from parents or guardians. If the need for consent was waived by the ethics committee, please include this information.

If you are reporting a retrospective study of medical records or archived samples, please ensure that you have discussed whether all data were fully anonymized before you accessed them and/or whether the IRB or ethics committee waived the requirement for informed consent. If patients provided informed written consent to have data from their medical records used in research, please include this information.

Answer: We don't have an ethical statement as this study is conducted based on publicly available datasets without any sensitive data and the study is not based on any survey from people
