# Supplementary material for: Offensive language detection in low resource languages: A use case of Persian language
Source: PLoS One. 2024 Jun 21;19(6):e0304166. doi: 10.1371/journal.pone.0304166 (PMC11192314; doi:10.1371/journal.pone.0304166)
Supplement: Attachment — Submitted filename: plos one review.docx [file pone.0304166.s001.docx]

Contribution: compare many models, present new architecture in hope of solving the problem with improved performance and an experiment. Persian Dataset annotation

Relevant literature: adequately cited and discussed w.r.t different languages and different models for the chosen task. However choices are only cursorily supported by literature

Presentation: good

Clarity: fair enough

Organization: fair enough(most revisions relate to this)

Reproducible results: yes, model seem adaptable

Purpose of model: well described/ supported by literature

Input/output and internal processing: fairly described

Illustrations: well made

Minor Revisions:

1. References are not an alphabetically ordered list
2. The English of write-up can be improved overall, headings should be in sentence case and punctuation marks at correct places.
3. The inclusion of web links in text is not a good idea, add in footnotes or references
4. All the sections and sub sections have to be numbered, right from beginning with introduction to conclusion
5. There is no discussion about future recommendations , not even a single line in the last section
6. Add percentage of achieved improvement in conclusion section
7. Wherever mention number of instances of dataset, use complete number: 520,000 instead of 520k
8. At the end of introduction section , a paragraph that describes the organization of the rest of the paper is required (outline of the study)
9. Main contributions in the introduction section has to be numbered list rather than bullet points
10. The figure elaborating the research methodology needs revision, like in preprocessing module all steps need to be connected(right now they looked stacked) , also inclusion of input and output labels/evaluation methods is nowhere except mentioning that tweets are the input, also there is no mention of datasets in the figure
11. add separate tables for names of models involved in various sub sections of methodology section, along with their parameters and specs
12. Can I ask why this split of 90-10? I mean with such copious amount of data, is it needed? Is it not too biased split?
13. Preprocessing section contains some vague text in language other than English
14. A diagram of data annotation process can be added to illustrate it in dataset section
15. A diagram of stacking ensemble’s working, as given by the algorithm, is needed
16. the algorithm need line numbers in it and should be explained with reference of line numbers in the paragraph above it.
17. There has to be a clear mention of the evaluation metrics to be used in the methodology or experiment section (right now its slightly mentioned in the start of results section)
